# Clinical decision support of therapeutic drug monitoring of phenytoin: measured versus adjusted phenytoin plasma concentrations

**DOI:** 10.1186/1472-6947-12-7

**Published:** 2012-02-14

**Authors:** Matthew D Krasowski, Louis E Penrod

**Affiliations:** 1 Department of Pathology, University of Iowa Hospitals and Clinics, Iowa City, IA 52242, USA; 2 Department of Physical Medicine and Rehabilitation, University of Pittsburgh, Pittsburgh, PA 15213, USA; 3 Baptist Health South Florida, Miami, FL 33176, USA

## Abstract

**Background:**

Therapeutic drug monitoring of phenytoin by measurement of plasma concentrations is often employed to optimize clinical efficacy while avoiding adverse effects. This is most commonly accomplished by measurement of total phenytoin plasma concentrations. However, total phenytoin levels can be misleading in patients with factors such as low plasma albumin that alter the free (unbound) concentrations of phenytoin. Direct measurement of free phenytoin concentrations in plasma is more costly and time-consuming than determination of total phenytoin concentrations. An alternative to direct measurement of free phenytoin concentrations is use of the Sheiner-Tozer equation to calculate an adjusted phenytoin that corrects for the plasma albumin concentration. Innovative medical informatics tools to identify patients who would benefit from adjusted phenytoin calculations or from laboratory measurement of free phenytoin are needed to improve safety and efficacy of phenytoin pharmacotherapy. The electronic medical record for an academic medical center was searched for the time period from August 1, 1996 to November 30, 2010 for patients who had total phenytoin and free phenytoin determined on the same blood draw, and also a plasma albumin measurement within 7 days of the phenytoin measurements. The measured free phenytoin plasma concentration was used as the gold standard.

**Results:**

In this study, the standard Sheiner-Tozer formula for calculating an estimated (adjusted) phenytoin level more frequently underestimates than overestimates the measured free phenytoin relative to the respective therapeutic ranges. Adjusted phenytoin concentrations provided superior classification of patients than total phenytoin measurements, particularly at low albumin concentrations. Albumin plasma concentrations up to 7 days prior to total phenytoin measurements can be used for adjusted phenytoin concentrations.

**Conclusions:**

The results suggest that a measured free phenytoin should be obtained where possible to guide phenytoin dosing. If this is not feasible, then an adjusted phenytoin can supplement a total phenytoin concentration, particularly for patients with low plasma albumin.

## Background

Recent reports by the Institute of Medicine (IOM) have publicized the risk of medical errors inherent in America's healthcare system [1,2]. To substantially improve the quality of care, the IOM has called for computerized physician order entry (CPOE) coupled with clinical decision support systems (DSS) [2]. Such a combination has been shown to reduce medical errors [3] and speed adoption of new patterns of practice [4] as prime examples of improved quality of care. Still more recent reports indicate that the processes by which CPOE systems are implemented are the key to success or failure in reducing errors [5-7]. The same diligence in implementation and ongoing review are needed for clinical DSS to effect change positively.

A significant challenge in clinical care is administration of phenytoin (PHT) to control seizures. Drug levels that are low may not control the seizures adequately; while drug levels that are too high can result in toxic effects. PHT dosing is challenging because the drug exhibits non-linear pharmacokinetics, zero-order elimination, and a multitude of drug-drug interactions [8-11]. For this reason, drug levels are measured to optimize dosing, with the usual therapeutic range for plasma total PHT concentration (PHT_total_) considered to be 10-20 mg/L [12]. PHT is also highly bound to plasma proteins. It is the free, or unbound, portion of the drug that is biologically active and which causes both therapeutic and toxic effects [13]. The therapeutic range for free PHT plasma concentrations (PHT_free_) is generally considered to be 1-2 mg/L (i.e., PHT_total _divided by 10 or PHT_total/10_), using an estimated 10% free fraction of PHT [12].

One factor that causes significant variation in PHT plasma protein binding is a low plasma albumin level (hypoalbuminemia), leading to an increased free PHT fraction, although other factors such as uremia or drug-drug interactions (e.g., inhibition of phenytoin metabolism by valproic acid) can also alter the free fraction of PHT [14,15]. In this situation, the usual PHT_total _assay, which measures both bound and free portions together, may provide discrepant results relative to the PHT_free _concentration [8,16]. Equations to estimate an adjusted free PHT (PHT_adj_free_) using a PHT_total _value exist to better approximate dose and gauge clinical efficacy. A common method of predicting the effect of albumin plasma concentrations on PHT level is the Sheiner-Tozer equation [14,15], which can be used to calculate PHT_adj_free_. If a DSS using this method indicates that the PHT_adj_free _is outside the desired target concentration range in a given individual, then PHT dose may be adjusted by the clinician and, in addition, the clinician may decide to monitor PHT_free _directly. In this study, we perform a large retrospective study to determine how well the PHT_adj_free _concentrations compare to direct measurement of PHT_free_.

## Results and Discussion

There were a total of 1,753 datapoints from 756 patients that had simultaneous determination of PHT_total _and PHT_free _and also a plasma albumin measured within 7 days of the PHT measurements. As shown in Table 1, the patient population studied was mostly 15 years or older (n = 701 out of 756). Roughly equal numbers of patients were being administered monotherapy with phenytoin for seizure control (n = 386) as compared to being prescribed one or more additional anti-epileptic drugs in addition to phenytoin (n = 370). The most common co-administered anti-epileptic drugs were levetiracetam (167 patients), phenobarbital (51 patients), and valproic acid (44 patients) (Table 1). At the time of blood draw for the initial phenytoin drug level, 263 patients had documentation of seizure within 24 hrs while 493 patients did not.

**Table 1:** Study population

	Males	Females
**Total**	n = 422	n = 334

**Age**		
**Average ± SD**	52.4 ± 20.9	54.8 ± 21.8
**0-12 months**	n = 12	n = 8
**1-14 years old**	n = 22	n = 13
**15-30 years old**	n = 33	n = 24
**31-59 years old**	n = 192	n = 142
**60-79 years old**	n = 138	n = 108
**80 years old or older**	n = 25	n = 39

**Adult inpatient (not ICU^a^)**	n = 287	n = 248
**Pediatric inpatient (not ICU)**	n = 19	n = 10
**ICU**	n = 88	n = 59
**Emergency room**	n = 5	n = 5
**Primary care clinic**	n = 4	n = 4
**Other outpatient clinic**	n = 15	n = 8

**Monotherapy with phenytoin**	n = 200	n = 171
**One additional anti-epileptic drugs^b^**	n = 176	n = 129
**Two additional anti-epileptic drugs^b^**	n = 38	n = 29
**Three or more additional anti-epileptic drugs^b^**	n = 8	n = 5

**No seizures within 24 hrs of phenytoin drug level**	n = 265	n = 228
**Seizure(s) within 24 hrs of phenytoin drug level**	n = 157	n = 106

^a ^*ICU *intensive care unit

^b ^Number of patients administered other anti-epileptic drugs: carbamazepine, 15; clonazepam, 5; diazepam, 10; felbamate, 9; lacosamide, 16; lamotrigine, 30; levetiracetam, 167; lorazepam, 56; pentobarbital, 14; phenobarbital, 51; primidone, 6; topiramate 19; valproic acid 44; zonisamide, 13

**Figure 1:**
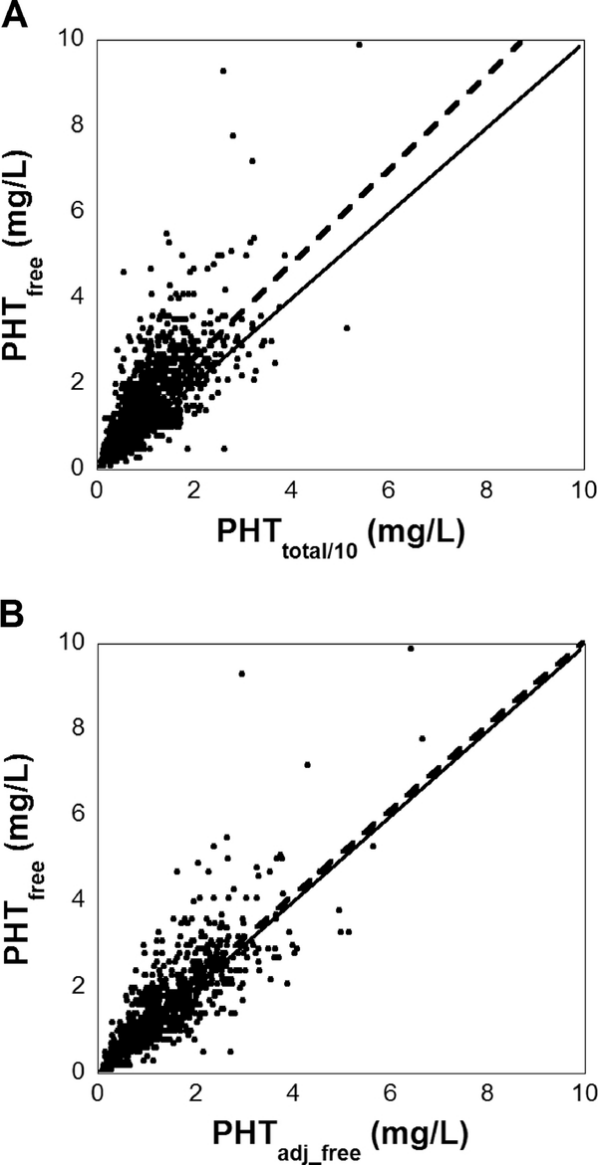
**Correlation of total, adjusted, and free phenytoin plasma concentrations**. (**A**) Correlation of PHT_free _versus PHT_total/10 _for 756 patients is shown. For patients who had multiple phenytoin measurements, only the chronologically first set of PHT_free _and PHT_total/10 _measurements is plotted. The solid line is the line of identity, and the dashed line is from linear regression: PHT_free _= 0.385 + 1.104 * PHT_total/10 _(R^2 ^= 0.51). The 95% confidence intervals of the intercept is (0.355, 0.415) and for the slope is (1.045,1.163). (**B**) Correlation of PHT_free _versus PHT_adj_free _is shown using same source of patient data as in (**A**). The dashed line is from linear regression: PHT_free _= 0.223 + 0.980 * PHT_adj_free _(R^2 ^= 0.62). The 95% confidence intervals of the intercept is (0.129, 0.317) and for the slope is (0.926, 1.034)

Figures 1A and 1B show scatterplots of PHT_free _versus PHT_total/10 _and PHT_free _versus PHT_adj_free_,, respectively, using only the initial laboratory data for patients (i.e., not including repeat measurements for patients). The Pearson coefficient for the correlations between PHT_total/10 _and PHT_free _was 0.72 and for PHT_free _and PHT_adj_free _was 0.79. The slope of the regression line for the relationship between PHT_free _versus PHT_total/10 _was statistically different than the line of identity (slope = 1) (95% confidence interval: 1.045-1.163, P < 0.05 for comparison of slope to 1). In contrast, the slope of the regression line for the relationship between PHT_free _versus PHT_adj_free _was not statistically different from 1 (95% confidence interval: 0.926-1.034). Additional file 1: Figure S1 (found in Additional file 1) shows plots similar to Figure 1A and 1B except using all laboratory data, including repeated measurements (i.e., using all 1,753 datapoints from 756 patients). These present very similar relationships to that seen in Figure 1A and 1B. Additional file 1: Figure S2 presents Bland-Altman (difference) plots of the data in Figure 1B, both by absolute (Additional file 1: Figure S2A) and percent bias (Additional file 1: Figure S2B).

**Figure 2:**
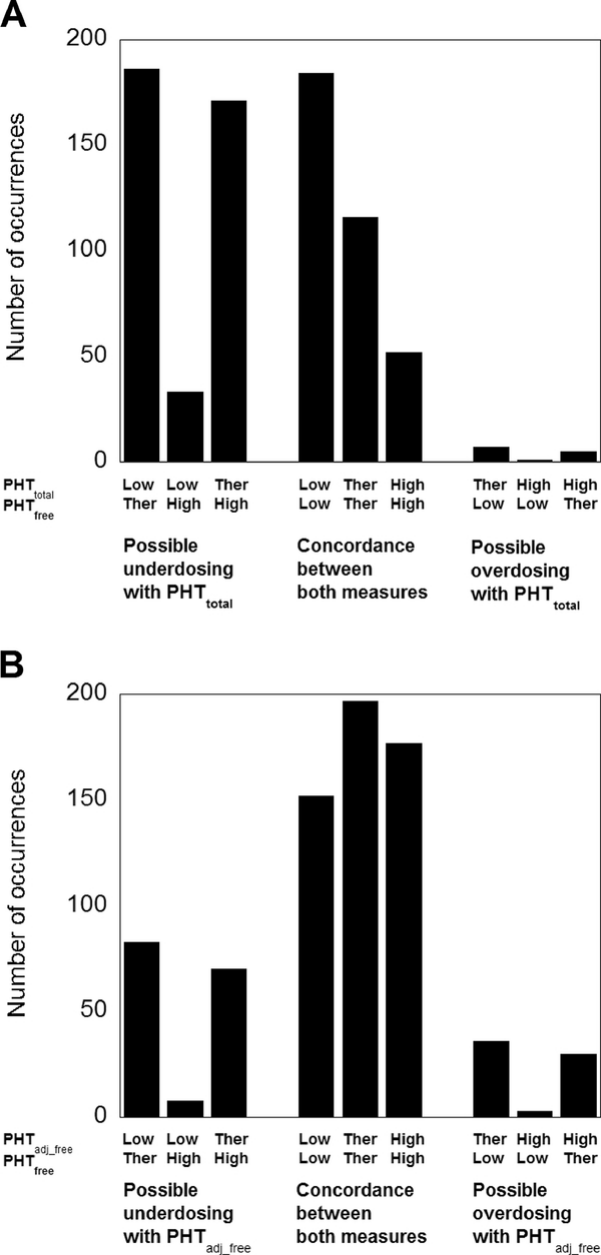
**Clinical decision using total phenytoin or adjusted phenytoin compared to measured free phenytoin**. (**A**) The data is derived from three-by-three contingency tables comparing grouping of PHT_total _and PHT_free _into lower than therapeutic reference range (L), within therapeutic reference range (T), and higher than therapeutic reference range (H). For the three bar graphs on the left, PHT_total _would tend to lead to underdosing relative to the PHT_free_. For the three bar graphs in the center, PHT_total _and PHT_free _are concordant. For the three bar graphs on the left, PHT_total _would tend to lead to overdosing relative to PHT_free_. For patients who had multiple phenytoin measurements, only the chronologically first set of concentrations available is plotted. The data is from 756 patients. (**B**) Similar design as in (**A**) except that PHT_adj_free _(using the Sheiner-Tozer equation) is compared to PHT_free_. The data is from 756 patients

Although the PHT_adj_free _provides a higher Pearson correlation to PHT_free _than PHT_total/10_, the question is whether a clinician is more likely to make a different decision when presented with PHT_adj_free _as opposed to PHT_total_. This was investigated through the use of contingency tables. Three-by-three contingency tables were constructed comparing grouping of results for PHT_total _and PHT_free _with respect to their therapeutic ranges (10-20 mg/L for total phenytoin; 1-2 mg/L for free phenytoin). As Figure 2A and Additional file 1: Figure S3A shows, PHT_total _frequently is in a lower category than PHT_free _(e.g., PHT_total _below its therapeutic range but PHT_free _within or above its reference range), which could lead to clinical overdosing of the patient if PHT_total _and not PHT_free _were used as the basis to guide dosing. The converse situation (PHT_free _in a lower category than PHT_total_) was uncommon. Overall, PHT_free _and PHT_total _were concordant with respect to therapeutic category less than 50% of the time (43.1% for all datapoints and 46.6% when excluding repeated measurements).

On the other hand, three-by-three contingency tables showed that PHT_adj_free _had improved concordance, relative to PHT_total_, to PHT_free _with respect to therapeutic category (Figure 2B, Additional file 1: Figure S3B). Similar to the analysis between PHT_total _and PHT_free_, PHT_adj_free _was more frequently in a lower category to PHT_free _than in a higher category. However, PHT_free _and PHT_adj_free _were concordant nearly 70% of the time (68.7% for all datapoints and 69.6% when excluding repeated measurements), statistically superior to PHT_total _(Fisher's exact test < 0.001).

The concordance data was also broken down into patients who did or did not have documented seizures within 24 hours of the blood draw for phenytoin drug level (Additional file 1: Figure S4) and those on phenytoin monotherapy for seizure therapy versus those also being treated with additional anti-epileptic drugs (Additional file 1: Figure S5). PHT_free _and PHT_adj_free _were concordant 73.2% for patient without recent seizures but only 56.6% for those who had seizures within 24 hours (Fisher's exact test < 0.001). In contrast, PHT_free _and PHT_adj_free _were concordant 69.9% for patients on phenytoin monotherapy and 69.3% for patients on polytherapy for seizure control (Fisher's exact test > 0.05).

We also looked at the three-by-three contingency table data to see how stable phenytoin measurements were for patients who had multiple phenytoin measurements over time. In particular, we compared how often, for a given patient, the temporally next phenytoin measurement fell in the same category in the three-by-three table as the previous set of measurements. For the data comparing PHT_total _with PHT_free_, the next consecutive set of phenytoin measurements agreed 53.7% of the time with the previous measurements (529 out of 997 measurements). For the data comparing PHT_adj_free _with PHT_free_, the next consecutive set of phenytoin measurements agreed 46.9% of the time with the previous measurements (468 out of 997 measurements). These data may reflect the predominantly inpatient population studied, where shifts in phenytoin dosing and also changes in other factors (e.g., concomitant) were needed for patients with unstable clinical status.

**Figure 3:**
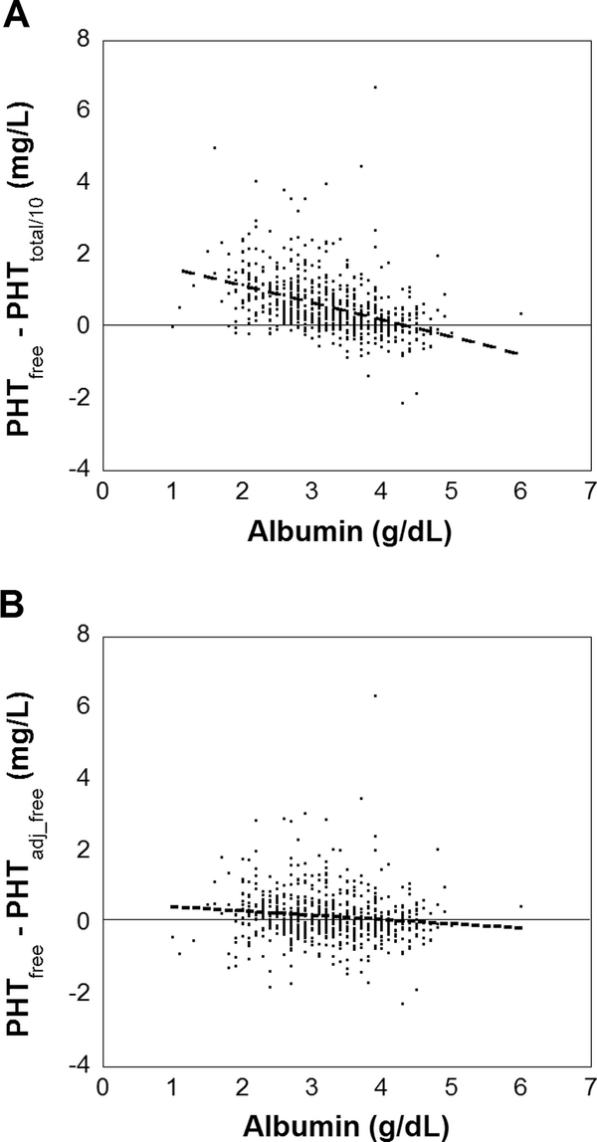
**Variation of total phenytoin, adjusted phenytoin, and free phenytoin with respect to plasma albumin concentration**. (**A**) Variation of the difference between PHT_total/10 _and PHT_free _with respect to plasma albumin concentration. All data is from chronologically first phenytoin measurements in 756 patients. The dashed line is from linear regression: [PHT_total/10 _- PHT_free_] = -2.136 + 0.477 * [albumin concentration] (R^2 ^= 0.19). The 95% confidence intervals of the intercept is (-2.37, -1.90) and for the slope is (0.406, 0.549). (**B**) Variation of the difference between PHT_free _and PHT_adj_free _with respect to plasma albumin concentration using same source of patient data in (**A**). The dashed line is from linear regression: [PHT_free _- PHT_adj_free_] = 0.0838 + 0.0021*[plasma albumin] (R^2 ^= 0.004). The 95% confidence intervals of the intercept is (-0.138, 0.305) and for the slope is (-0.0649, 0.0691)

The difference between PHT_total/10 _and PHT_free _was also examined in relation to plasma albumin concentration (Figure 3A, Additional file 1: Figure S6A). The discrepancy between PHT_free _and PHT_total/10 _is most pronounced at low plasma albumin concentrations, where the ratio of PHT_free _to PHT_total _would be expected to be higher. However, there are clearly many examples of marked discrepancies between PHT_free _and PHT_total/10 _even when the plasma albumin concentrations is within the age-specific reference range. PHT_free _is generally greater than PHT_total/10 _throughout all ages with examples of patients showing differences of > 2 mg/L evident throughout all age groups. The slope of the regression line in Figure 3Awas significantly different from 0 (i.e., null hypothesis of no relationship; 95% confidence interval; -2.37 to -1.90; P < 0.05). In contrast, the difference between PHT_free _and PHT_adj_free _shows little relationship with respect to plasma albumin concentration (Figure 3B, Additional file 1: Figure S6B), with the slope of the regression line in Figure 3B showing no significant difference from 0 (95% confidence interval: -0.0649 to 0.0691).

**Figure 4:**
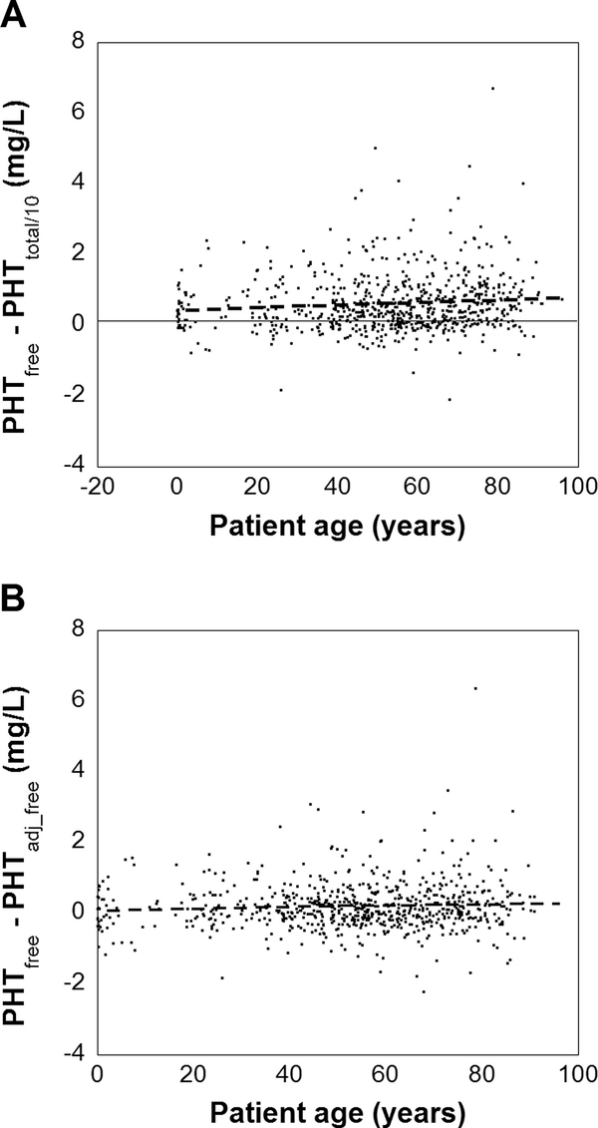
**Variation of total phenytoin, adjusted phenytoin, and free phenytoin with respect to patient age**. (**A**) Variation of the difference between PHT_total/10 _and PHT_free _with respect to patient age. All data from chronologically first phenytoin measurements in 756 patients. The dashed line is from linear regression: [PHT_total/10 _- PHT_free_] = -0.394 + 0.0038*(patient age) (R^2 ^= 0.01). The 95% confidence intervals of the intercept is (-0.464, -0.372) and for the slope is (-0.056, 0.064). (**B**) Variation of the difference between PHT_free _and PHT_adj_free _with respect to patient age is shown using same source of data as in (**A**). The dashed line is from linear regression: [PHT_free _- PHT_adj_free_] = 0.0838 + 0.0021 * age (R^2 ^= 0.004). The 95% confidence intervals of the intercept is (-0.0072, 0.174) and for the slope is (-0.053, 0.057)

The difference between PHT_total/10 _and PHT_free _was also examined in relation to patient age (Figure 4A, Additional file 1: Figure S7A), which revealed no statistically significant difference of the slope of the regression line in Figure 4A from 0 (95% confidence interval: -0.056 to 0.064). A similar finding was noted between the difference between PHT_free _and PHT_adj_free _(Figure 4B, Additional file 1: Figure S7B). The slope of the regression line in Figure 4B showed no statistically significant difference from 0 (95% confidence interval: -0.053 to 0.057).

**Figure 5:**
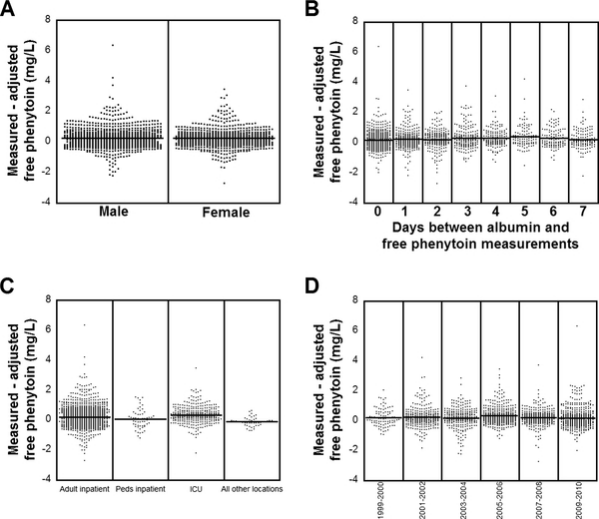
**Variation of the difference of PHT_free _and PHT_adj_free _with respect to various independent variables. (A) Distribution of the difference between PHT_free _and PHT_adj_free _separated between males and females**. Each dot represents a single timepoint of data with a total of 1,020 datapoints from male patients and 733 datapoints from female patients. The solid horizontal lines indicate the mean difference. There was no significant difference between males and females (unpaired *t*-test > 0.05). (**B**) Distribution of the difference between PHT_free _and PHT_adj_free _separated by the days in between the albumin and total phenytoin measurements (that allowed for PHT_adj_free _calculation). Each dot represents a single timepoint of data with a total of 1,753 datapoints (559 datapoints for 0 days difference between albumin and PHT_total _determination, 290 for 1 day difference, 215 for 2 days difference, 190 for 3 days difference, 152 for 4 days difference, 103 for 5 days difference, 112 for 6 days difference, and 132 for 7 days difference). The solid horizontal lines indicate the mean difference. There was no significant difference across days between albumin and phenytoin measurements (ANOVA *P *value > 0.05). (**C**) Distribution of the difference between PHT_free _and PHT_adj_free _separated into categories of patient location at time of laboratory ordering of PHT_total _and PHT_free_. 'Adult inpatient' and 'Peds inpatient' encompass all inpatient locations excluding intensive care units (ICUs), with adult defined as age 15 or older. 'All other locations' encompasses outpatient clinics, emergency room, and scheduled draws at hospital phlebotomy stations. (**D**) Distribution of the difference between PHT_free _and PHT_adj_free _separated into years in which the laboratory measurements were performed. There was a significant effect of patient location at time of PHT measurements (ANOVA *P *value < 0.0001)

A series of analyses were also done to try to understand what additional factors might influence how well PHT_adj_free _predicts PHT_free_, using plots of the difference between PHT_free _and PHT_adj_free _and various independent variables (Figure 5; note that the statistical analyses in 5A-5D were four separate procedures). There is little influence of patient gender (Figure 5A) or days between albumin and PHT_free_/PHT_total _measurements (Figure 5B) on the difference between PHT_free _and PHT_adj_free_. There was, however, a significant effect of patient location at time of PHT measurements (Figure 5C), with the deviation between PHT_free _and PHT_adj_free _highest in adult inpatient (non-ICU) and ICU units. In the inpatient settings, PHT_free _was significantly greater than PHT_adj_free_. PHT_free _- PHT_adj_free _did not vary significantly with the year in which the phenytoin measurements were performed (Figure 5D), suggesting that changes in clinical laboratory instrumentation and assays over the years of the retrospective analysis did not cause any changes in PHT_free_, PHT_total_, or plasma albumin concentrations that might systematically impact the relation of PHT_free _to PHT_adj_free_.

Since its initial development, the Sheiner-Tozer equation has been widely used to assist therapeutic drug monitoring of phenytoin [14,15]. It is even included in the MedMath module of Epocrates™ software (registered trademark of Epocrates, Inc., San Mateo, CA, USA,). Despite this widespread use, there has been considerable controversy over whether inaccuracies in the model justify its use or not. Some authors have argued that since PHT_free _levels may not be readily available, the adjustment in cases of known hypoalbuminemia provides better guidance in dosing than the total PHT level obtained in the usual assay used for therapeutic drug monitoring [9,15,17]. In a population of "critically ill neurosurgical patients", Mlynarek et al. concluded that the Sheiner-Tozer equation provided "an unbiased, precise clinical estimate" in cases where the PHT_free _level "is unavailable or impractical" [17]. For rural clinics in sub-Saharan Africa where malnutrition and AIDS are frequent, Fedler and Stewart concluded that the corrected value should be reported rather than the total phenytoin [9]. On the other hand, two reports from university hospital settings concluded that because of the inaccuracies of the model, the Sheiner-Tozer equation should not be used [14,18]. Other studies have recommended that PHT_total _not be used at all, and that PHT_free _alone be used for drug monitoring of PHT [19].

In our study, PHT_adj_free _provides a better estimate of PHT_free _(relative to reference range) than PHT_total_. Previous studies have focused on more limited sample sizes and patient populations [14,17,18,20]. Our study included a population of mainly adults in the inpatient setting, including patients with refractory epilepsy and/or who were on multiple other anti-epileptic medications in addition to phenytoin. The linear regression lines relating PHT_free _to PHT_adj_free _have a slope close to 1 with only a slight negative bias (~0.2-0.3 mg/L) of PHT_adj_free _relative to PHT_free_. However, we did demonstrate that the greatest bias between PHT_free _and PHT_adj_free _was seen in hospital inpatients, possibly due to other factors (e.g., concomitant drugs, organ failure) that can impact PHT pharmacokinetics.

In the university hospital setting, the cost to the clinical laboratory to perform the PHT_free _assay can be almost twice the cost of the PHT_total _assay. In addition, the PHT_free _assay process includes an extra ultra-filtration step that requires centrifugation [13,21]. Because of these extra steps, and for quality control, PHT_free _assays for a given day may be held and run in one or more batches during the day to limit labor-intensive steps. This contrasts with PHT_total _levels that may run throughout the day on automated instrumentation without need for separate processing steps. Thus, there may be a delay in receiving the PHT_free _results compared to the availability of the PHT_total _result on the same sample. In the case of a smaller hospital, where it may be longer until the batch of PHT_free _samples is accumulated and processed, or if PHT_free _levels are sent to a reference lab instead of done in house, the time disparity could be even greater. However, the added costs of the PHT_free _assay should be assessed in context with the risks of suboptimal phenytoin dosing (e.g., poor seizure control, toxicity, etc.).

Most of the "rule of thumb" equations in common use presume that the clinician is doing the calculation by hand or calculator, using a few readily available results to derive additional knowledge not directly reported. A modern electronic health record (EHR) with integrated DSS can provide added value for the clinician by doing these calculations automatically and posting the results, saving time and eliminating errors in calculation. One of the authors has taken this approach at the University of Pittsburgh Medical Center (UPMC) by developing and implementing rules to provide Anion Gap, Adjusted Sodium for hyperglycemia, and Adjusted Calcium for hypoalbuminemia, in addition to an Adjusted Phenytoin Rule. In all cases, interpretive data is attached to the result to further assist the clinician in decision making. Education of clinicians is important to emphasize the difference and limitations of both calculated and directly measured parameters.

There are several main limitations to the analysis presented in this paper. First, the patient population contains far more adults than pediatric patients. Consequently, the results are mostly applicable to adult patients. Future studies targeted at children, especially very young children, are needed. Second, the majority of datapoints arise from patients in inpatient units (including intensive care units) with lesser availability of datapoints arising from patients in outpatient clinics or the emergency room. However, the sample size of this study exceeds that of previous studies and contains a patient population that likely is similar to that analyzed by clinical laboratories at many academic medical centers, and has produced results comparable to other similar studies of academic medical center patient populations [14,18]. Lastly, although nutritional status was not examined in detail, it is likely that most patients in the study were well-nourished and thus the findings are most applicable to other well-nourished populations.

Addressing the question of whether a better model than the Sheiner-Tozer equation could be implemented is the subject of additional analysis and development currently underway. The analysis reported here, along with the capabilities of the EHR and DSS, suggest additional possibilities to enhance the medical knowledge available to clinicians at the point of care. In addition to hypoalbuminemia, there are other factors that can influence binding of PHT and alter the free PHT fraction [14]. With a "rule of thumb" type calculation, it is prohibitive to track these additional factors and perform the calculations they would entail by hand or using a non-programmable calculator. However, given the extensive data available electronically in the EHR and the logic capabilities of a modern DSS, it may become practical to implement far more complex models than those traditionally used in clinical practice. With knowledge discovery tools, the data available in the EHR database provide a substrate for increasingly sophisticated models. The development and testing of a model that predicts free phenytoin better than done by the Sheiner-Tozer model is the focus of additional research by the authors.

## Conclusions

In this study, the standard formula for calculating an estimated (adjusted) phenytoin level more frequently underestimates than overestimates the free phenytoin relative to the respective therapeutic ranges. Estimated free phenytoin concentrations predicted measured free phenytoin concentrations better than total phenytoin measurements, although there is considerable scatter in the data. The results suggest that a measured free phenytoin should be obtained where possible to guide phenytoin dosing. If this is not feasible, then an adjusted phenytoin can supplement a total phenytoin concentration, with concurrent education of physicians as to the limitations of the adjusted phenytoin prediction.

## Methods

The project had Institutional Review Board approval from the University of Iowa. The electronic medical record (Epic, Epic Systems Inc., Madison, WI, USA) was searched for the time period from 8/1/1996 to 11/30/2010 for patients with PHT_total _and PHT_free _determined on the same blood draw and also a plasma albumin determined within the past seven days of the phenytoin measurements, with PHT_total_, PHT_free_, plasma albumin concentration, patient age, location (e.g., inpatient, outpatient, etc.) and gender downloaded. Chart review was performed to ascertain other anti-epileptic medications used in addition to phenytoin, and also whether patients had seizures within 24 hrs of the phenytoin drug level. During the entire time period of the retrospective analysis, the University of Iowa Hospitals and Clinics central clinical laboratory used the same assays to measure plasma phenytoin and free phenytoin on Roche Diagnostics (Indianapolis, IN, USA) P module automated chemistry analyzers. The total phenytoin measurement was performed using the Roche Phenytoin CEDIA (cloned enzyme donor immunoassay) method according to manufacturer's instructions. The free phenytoin assay was the Siemens Syva Emit 2000 Phenytoin Assay, an immunoassay adapted for use on the Roche modular P analyzer [22]. Specifically, plasma specimens were first allowed to equilibrate to room temperature. Then 500 μL of sample was applied to the sample reservoir of an assembled Amicon micropartition system MPS-1 (Millipore, Billerica, MA, USA), making sure no air was trapped in the reservoir. The reservoirs were then capped and centrifuged at 3400 rpm for 20 min using a centrifuge equipped with an angle head rotor. The ultrafiltrate was then transferred to a sample cup and analyzed. The characteristics of the study population are summarized in Table 1.

The Sheiner-Tozer equation is expressed as:

PHT_adj_free _= [PHT_total_/{(0.2 × Albumin) + 0.1)}]/10, with PHT plasma concentrations in units of mg/L and albumin plasma concentrations (Albumin) in units of g/dL. This assumes an estimated free fraction of PHT of 10%.

For comparison analysis, PHT_free _was considered the reference method (comparator). Comparisons of whether PHT_total_, PHT_free_, and PHT_adj_free _results fell within the same range compared to their respective therapeutic ranges gave an indication of the accuracy of the prediction models. Chi-squared analysis of decision making and ANOVA were performed using SPSS version 13.0 (SPSS, Inc., Chicago, IL, USA). Linear regression and Pearson correlation analysis were carried out in EP Evaluator release 9 (Data Innovations, South Burlington, VT, USA). Statistical comparison of linear regression utilized two-tailed t tests in EP Evaluator.

## Competing interests

The authors declare that they have no competing interests.

## Authors' contributions

MDK and LEP were both involved in the study concept and design, analysis and interpretation of the data. MDK drafted the manuscript. Both authors have read and approved the final manuscript.

## Pre-publication history

The pre-publication history for this paper can be accessed here:

http://www.biomedcentral.com/1472-6947/12/7/prepub

## Supplementary Material

Additional file 1 **Figure S1 (correlation of PHT_free _versus PHT_total/10 _and PHT_free _versus PHT_adj_free_), Figure S2 (Bland-Altman plots), Figure S3 (three-by-three contingency tables of PHT_total _and PHT_free_), Figure S4 (three-bv-three contingency tables of PHT_adj_free _and PHT_free _comparing those with recent seizures or no recent seizures), Figure S5 (three-bv-three contingency tables of PHT_adj_free _and PHT_free _comparing those on phenytoin monotherapy versus polytherapy with other anti-epileptic drugs), Figure S6 (effect of albumin concentration on free phenytoin estimation), and Figure S7 (effect of patient age on free phenytoin estimation)**. Click here for file
