# Supplementary material for: Clinical decision support of therapeutic drug monitoring of phenytoin: measured versus adjusted phenytoin plasma concentrations
Source: BMC Med Inform Decis Mak. 2012 Feb 14;12:7. doi: 10.1186/1472-6947-12-7 (PMC3312854; doi:10.1186/1472-6947-12-7)
Supplement: Additional file 1 — Figure S1 (correlation of PHTfree versus PHTtotal/10 and PHTfree versus PHTadj_free), Figure S2 (Bland-Altman plots), Figure S3 (three-by-three contingency tables of PHTtotal and PHTfree), Figure S4 (three-bv-three contingency tables of PHTadj_free and PHTfree comparing those with recent seizures or no recent seizures), Figure S5 (three-bv-three contingency tables of PHTadj_free and PHTfree comparing those on phenytoin monotherapy versus polytherapy with other anti-epileptic drugs), Figure S6 (effect of albumin concentration on free phenytoin estimation), and Figure S7 (effect of patient age on free phenytoin estimation). [file 1472-6947-12-7-S1.PDF]

Additional files

Clinical Decision Support of Therapeutic Drug Monitoring of Phenytoin: Measured Versus Adjusted Phenytoin Plasma Concentrations

Matthew D. Krasowski[1§],Louis E. Penrod[2,3]

[1]Department of Pathology, University of Iowa Hospitals and Clinics, Iowa City, IA, 52242, USA

[2]Department of Physical Medicine and Rehabilitation, University of Pittsburgh, Pittsburgh, PA, 15213, USA

[3]Baptist Health South Florida, Miami, FL 33176 (current address)

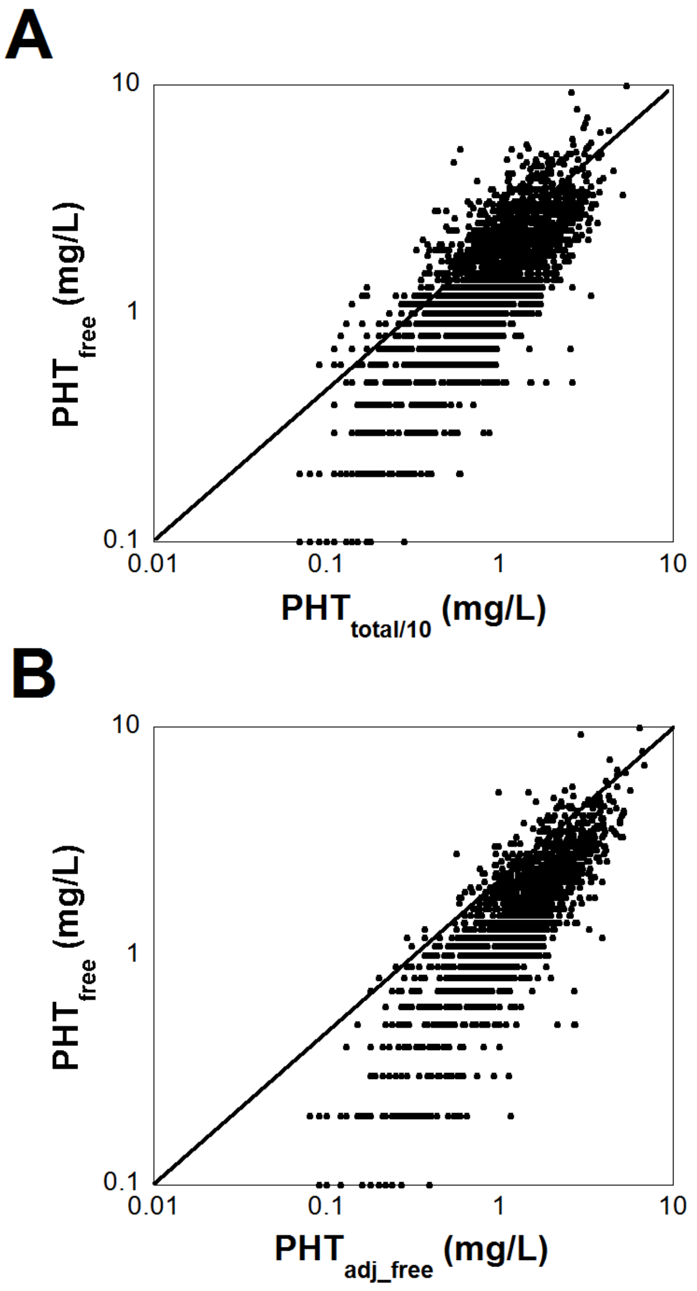

Additional figure 1

(A) Correlation of PHT_{free} versus $PHT_{total/10}$ using 1,753 datapoints from 756 patients is shown. Regression equation is: $PHT_{free} = 0.502 + 1.095 * PHT_{total/10}$ (R^2=0.49). (B) Correlation of PHT_{free} versus PHT_{adj_free} is shown using same source of patient data as in (A). Regression equation is: $PHT_{free} = 0.343 + 0.927 * PHT_{adj\_free}$ (R^2=0.64).

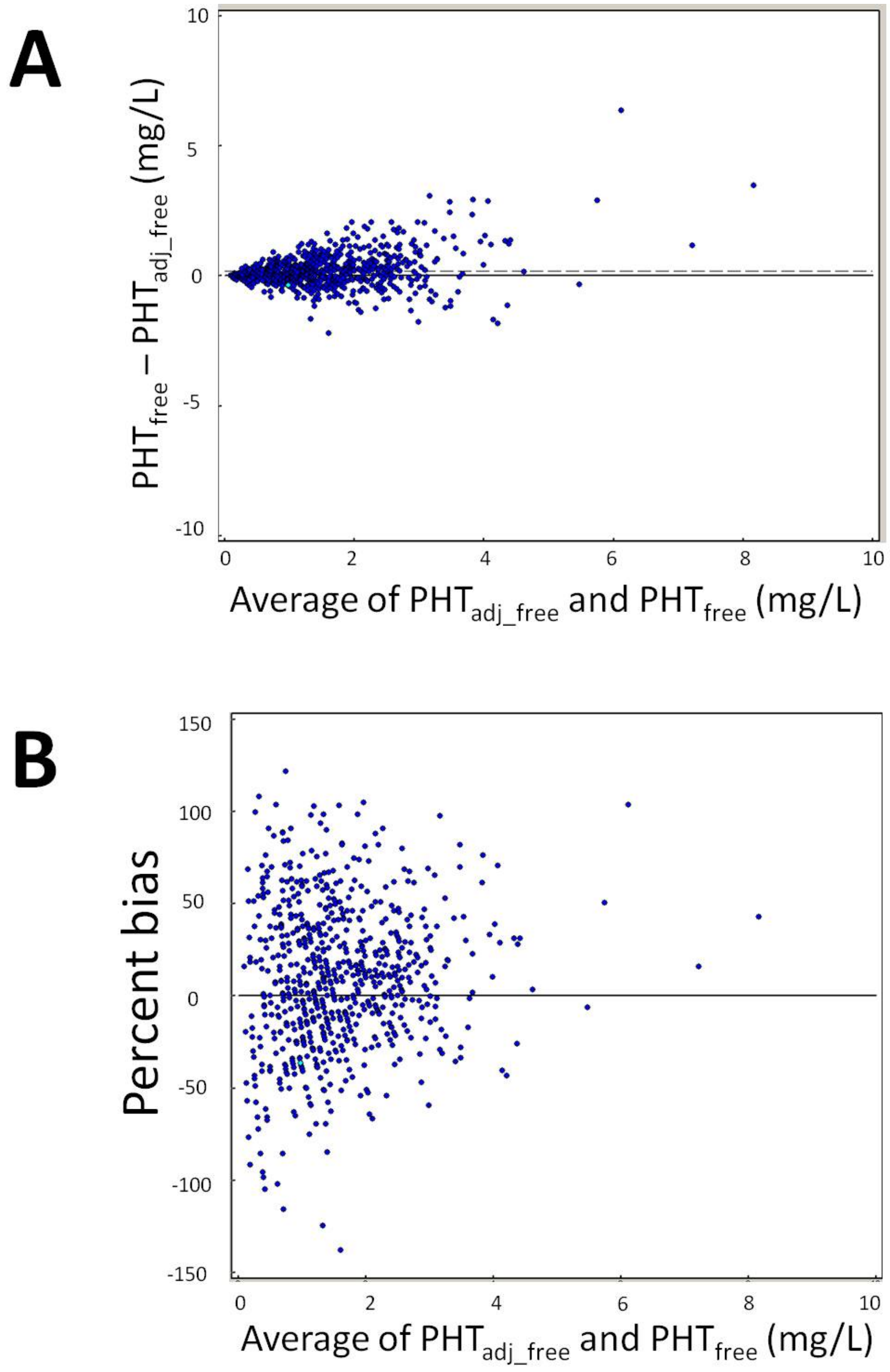

Additional figure 2

Bland-Altman (difference) plots of the relationship between PHT_{adj_free} and PHT_{free}, using data from 756 patients (repeated measurements excluded). (A) Absolute bias (PHT_{free} – PHT_{adj_free}) versus average of PHT_{adj_free} and PHT_{free}. (B) Percent bias.

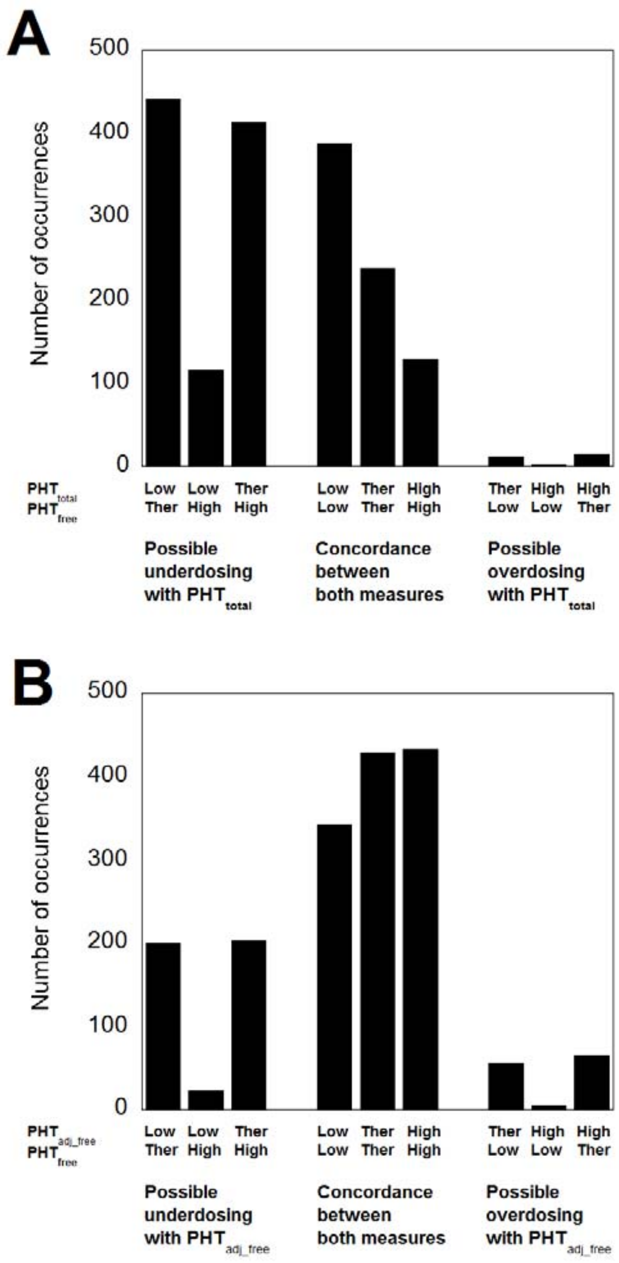

Additional figure 3

(A) The data is derived from three-by-three contingency tables comparing grouping of PHT_{total} and PHT_{free} into lower than therapeutic reference range (L), within therapeutic reference range (T), and higher than therapeutic reference range (H). The data is 1,753 datapoints from 756 patients. (B) Same design as in (A) except comparing PHT_{adj_free} with PHT_{free}.

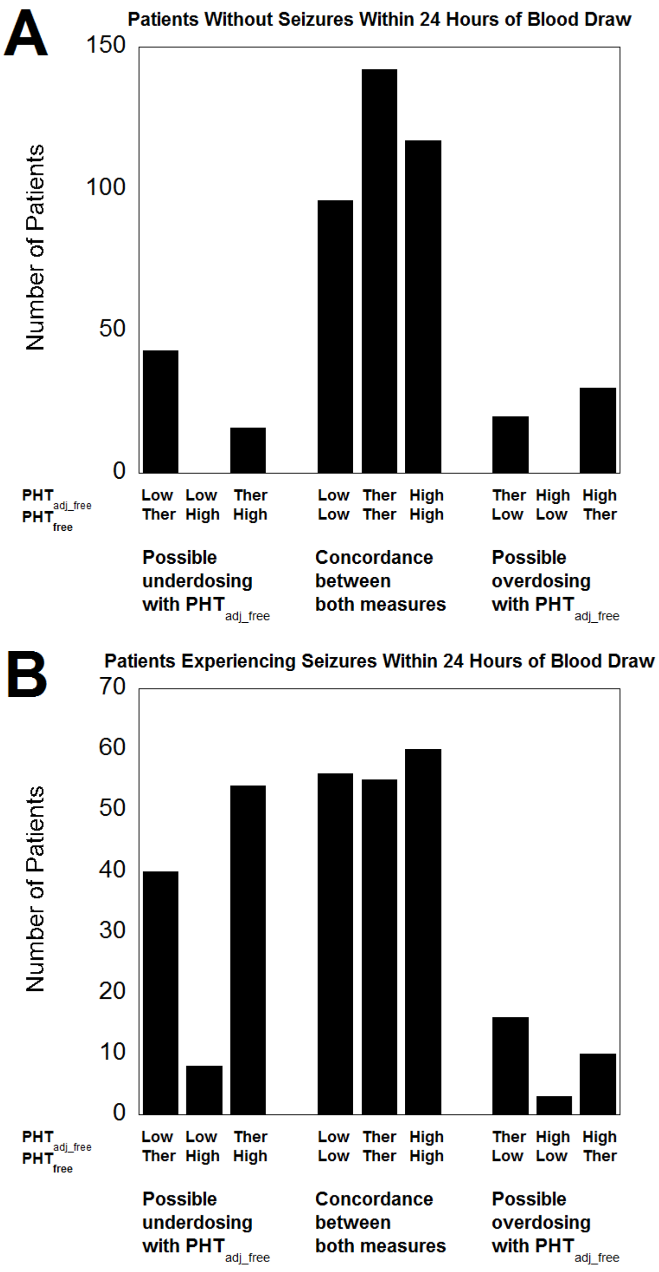

Additional figure 4

The data is derived from three-by-three contingency tables comparing grouping of PHT_{adj_free} and PHT_{free} into lower than therapeutic reference range (L), within therapeutic reference range (T), and higher than therapeutic reference range (H). (A) Patients who had no seizures documented within 24 hours of the blood draw for phenytoin drug level (n=492). (B) Patients who had at least one seizure documented within 24 hours of the blood draw for the phenytoin drug level (n=264). The data is from a total of 756 patients.

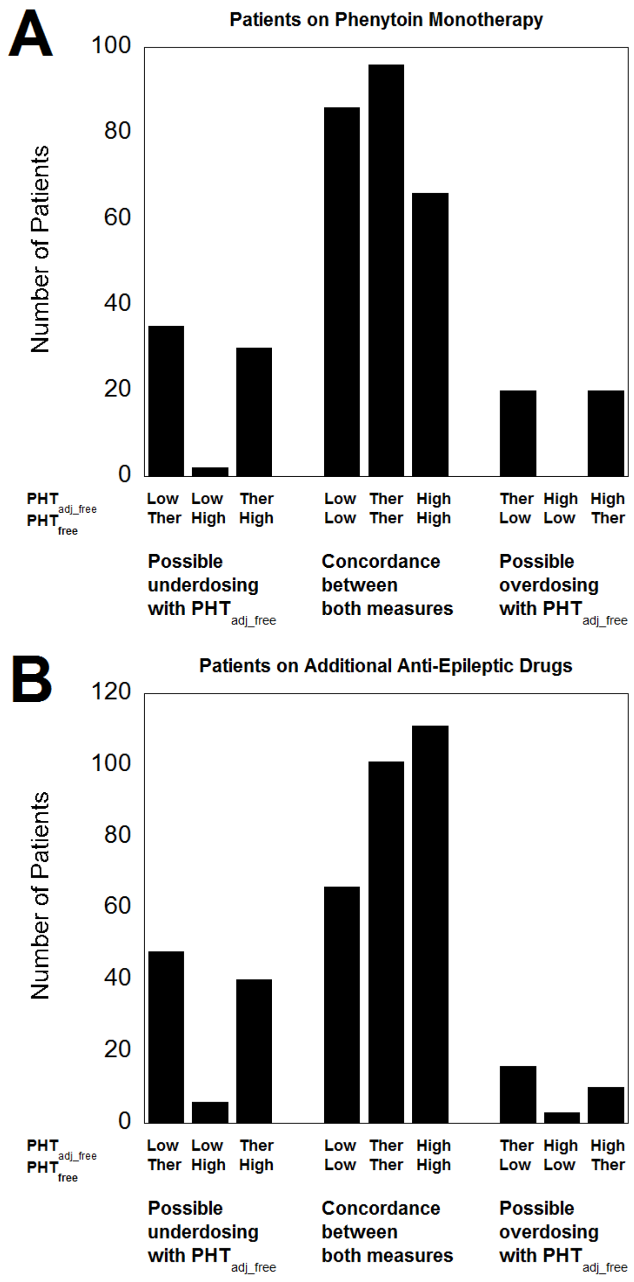

Additional figure 5

The data is derived from three-by-three contingency tables comparing grouping of PHT_{adj_free} and PHT_{free} into lower than therapeutic reference range (L), within therapeutic reference range (T), and higher than therapeutic reference range (H). (A) Patients who were on monotherapy with phenytoin (i.e., no additional anti-epileptic drugs) at time of the blood draw for phenytoin drug level (n=386). (B) Patients who were on at least one additional anti-epileptic drug at time of the blood draw for phenytoin blood level (n=370). The data is from a total of 756 patients.

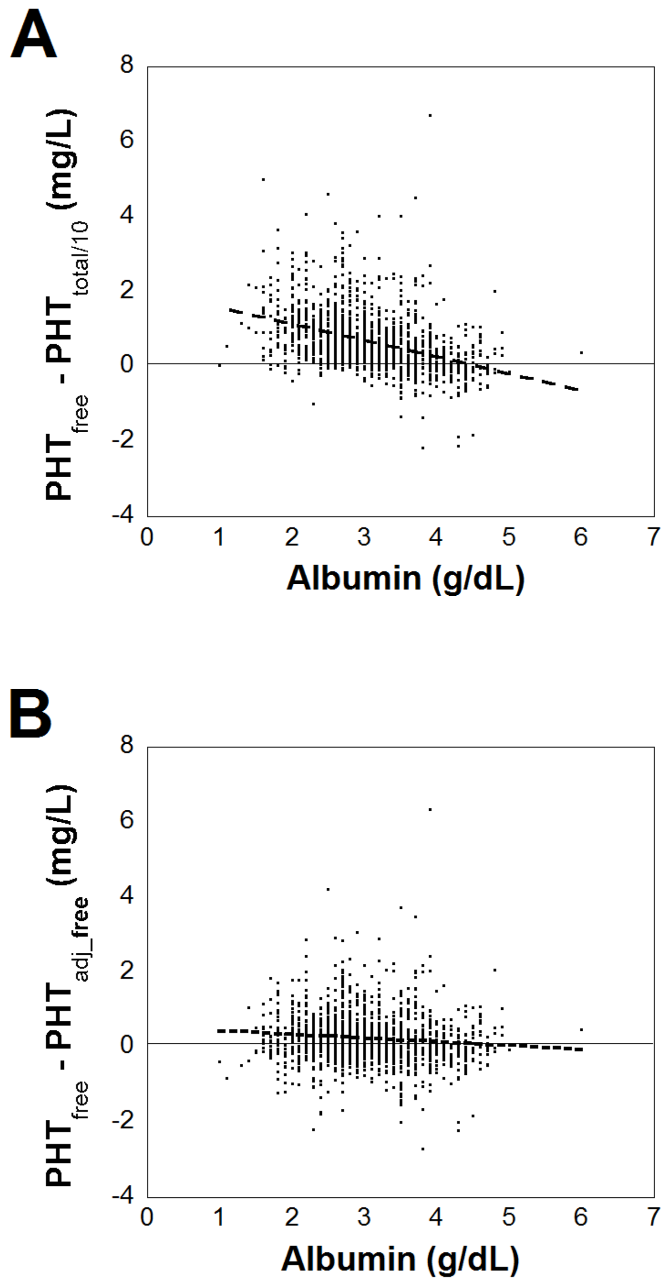

Additional figure 6

(A) Variation of the difference between $PHT_{total/10}$ and PHT_{free} with respect to plasma albumin concentration using 1,753 datapoints from 756 patients. The dashed line is from linear regression: [$PHT_{total/10}$ - PHT_{free}] = -2.054+0.446*[albumin concentration] (R^2=0.17). (B) Variation of the difference between PHT_{free} and PHT_{adj_free} with respect to plasma albumin patient age is shown using same source of data as in (A). The dashed line is from linear regression: [PHT_{free} - PHT_{adj_free}] = 0.145 + 0.0016*[plasma albumin] (R^2=0.002).

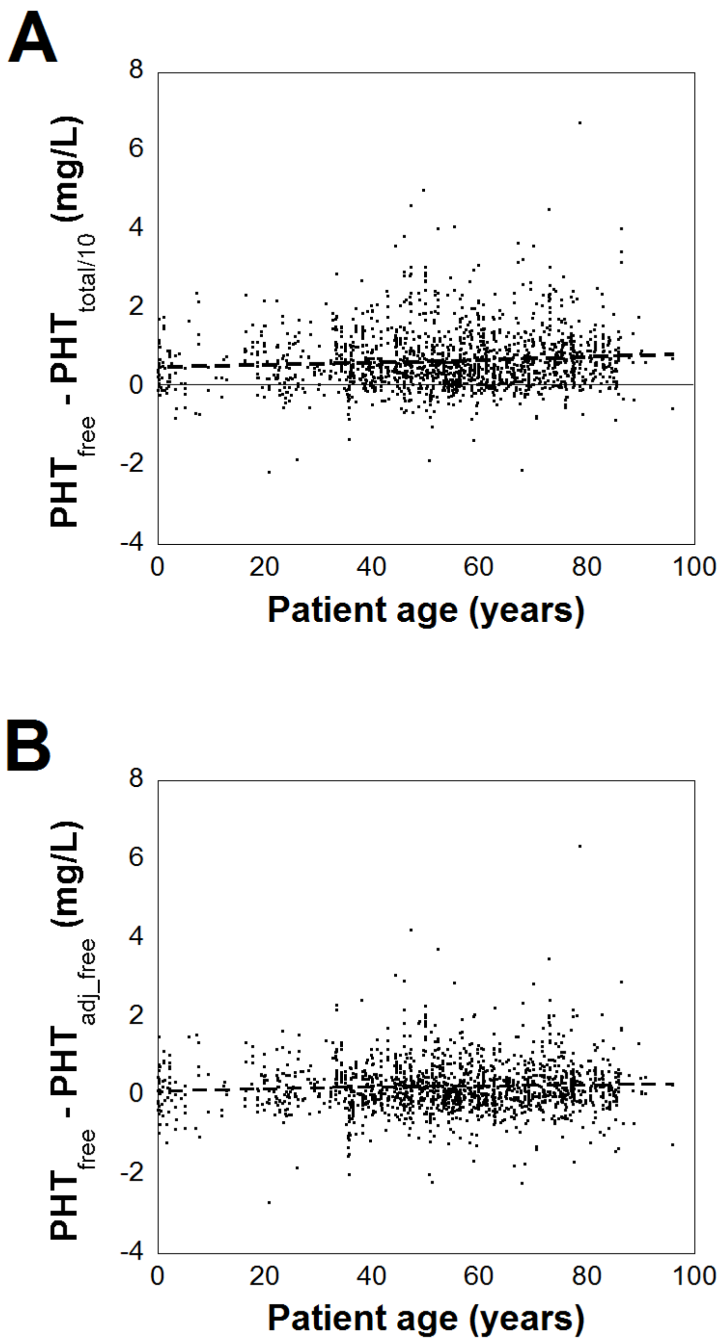

Additional figure 7

(A) Variation of the difference between $PHT_{total/10}$ and PHT_{free} with respect to patient age using 1,753 datapoints from 756 patients. The dashed line is from linear regression: [$PHT_{total/10}$ - PHT_{free}] = -0.520 + 0.0032 * (patient age) (R^2=0.01). (B) Variation of the difference between PHT_{free} and PHT_{adj_free} is shown using same source of data as in (B). The dashed line is from linear regression: [PHT_{free} - PHT_{adj_free}] = 0.145 + 0.0016 * age (R^2=0.002).
